# Retraction Note: Generation of long-chain fatty acids by hydrogen-driven bicarbonate reduction in ancient alkaline hydrothermal vents

**DOI:** 10.1038/s43247-025-02635-0

**Published:** 2025-08-07

**Authors:** Graham Purvis, Lidija Šiller, Archie Crosskey, Jupiter Vincent, Corinne Wills, Jake Sheriff, Cijo Xavier, Jon Telling

**Affiliations:** 1 School of Natural and Environmental Sciences, Newcastle University, Newcastle upon Tyne, NE1 7RU UK; 2 NEXUS, School of Engineering, Newcastle University, Newcastle upon Tyne, NE1 7RU UK

**Retraction to:** *Communications Earth & Environment* 10.1038/s43247-023-01196-4, published online 10 January 2024

The authors have retracted this article following concerns raised over the identification of fatty acids in the experimental products. Specifically, the gas chromatography-mass spectrometry data used to support the conclusion of abiotic generation of fatty acids under hydrothermal conditions contained several peaks which were misidentified. The identification was based on the use of a best fit algorithm but subsequent manual inspection indicates that these peaks do not represent evidence for the presence of fatty acids in the samples.

As the identification of fatty acids underpinned the principal conclusion of the contribution – that fatty acids had been generated in the experiments – this issue necessitated a retraction.

The authors acknowledge Tom McCollom for bringing the issue to their attention.

All authors agree with this retraction.

